# Genomic Analysis of Phylotype I Strain EP1 Reveals Substantial Divergence from Other Strains in the *Ralstonia solanacearum* Species Complex

**DOI:** 10.3389/fmicb.2016.01719

**Published:** 2016-10-26

**Authors:** Peng Li, Dechen Wang, Jinli Yan, Jianuan Zhou, Yinyue Deng, Zide Jiang, Bihao Cao, Zifu He, Lianhui Zhang

**Affiliations:** ^1^Guangdong Province Key Laboratory of Microbial Signals and Disease Control, State Key Laboratory for Conservation and Utilization of Subtropical Agro-Bioresources, Integrative Microbiology Research Centre, College of Agriculture, South China Agricultural UniversityGuangzhou, China; ^2^Guangdong Innovative and Entepreneurial Research Team of Sociomicrobiology Basic Science and Frontier Technology, College of Agriculture, South China Agricultural UniversityGuangzhou, China; ^3^Department of Vegetables, College of Horticulture, South China Agricultural UniversityGuangzhou, China; ^4^Plant Protection Research Institute Guangdong Academy of Agriculture SciencesGuangzhou, China; ^5^Institute of Molecular and Cell BiologySingapore, Singapore

**Keywords:** genome sequencing, comparative genomics, *Ralstonia solanacearum*, genome dynamics, virulence

## Abstract

*Ralstonia solanacearum* species complex is a devastating group of phytopathogens with an unusually wide host range and broad geographical distribution. *R. solanacearum* isolates may differ considerably in various properties including host range and pathogenicity, but the underlying genetic bases remain vague. Here, we conducted the genome sequencing of strain EP1 isolated from Guangdong Province of China, which belongs to phylotype I and is highly virulent to a range of solanaceous crops. Its complete genome contains a 3.95-Mb chromosome and a 2.05-Mb mega-plasmid, which is considerably bigger than reported genomes of other *R. solanacearum* strains. Both the chromosome and the mega-plasmid have essential house-keeping genes and many virulence genes. Comparative analysis of strain EP1 with other 3 phylotype I and 3 phylotype II, III, IV strains unveiled substantial genome rearrangements, insertions and deletions. Genome sequences are relatively conserved among the 4 phylotype I strains, but more divergent among strains of different phylotypes. Moreover, the strains exhibited considerable variations in their key virulence genes, including those encoding secretion systems and type III effectors. Our results provide valuable information for further elucidation of the genetic basis of diversified virulences and host range of *R. solanacearum* species.

## Introduction

*Ralstonia solanacearum*, a destructive bacterial pathogen that causes bacterial wilt diseases in over 400 plant species, has been recently ranked as the second most important bacterial plant pathogen (Mansfield et al., 2012). Accumulating evidences show that *R. solanacearum* is a species complex, a heterogeneous group of related but genetically distinct strains (Allen et al., 2005). *R. solanacearum* isolates collected from different regions of the world were usually remarkably different in many properties such as host range, pathogenicity, physiology, and even the genome sequences (Buddenhagen et al., 1962; Palleroni and Doudoroff, 1971; Hayward, 1991). Based on the genetic similarities of the internal transcribed spacer region, hypersensitive response and pathogenicity (*hrp*) gene *hrpB*, and endoglucanase, *R. solanacearum* species were grouped into 4 phylotypes (I–IV) (Fegan and Prior, 2005; Genin, 2010). Complete genome sequencing of *R. solanacearum* strain GMI1000 at the beginning of this century marked a significant advance in characterizing the molecular complexity governing both the pathogenicity and versatility of this complex of pathogens (Salanoubat et al., 2002; Genin and Boucher, 2004). Up to now, 54 *R. solanacearum* species have been sequenced (data from NCBI database; Sep. 2016); while most of these genome assemblies were in a “draft” status, the genomes of strains GMI1000 (phylotype I, France), YC45 (phylotype I, China), FQY_4 (phylotype I, China), PO82 (phylotype II, Mexico), CMR15 (phylotype III, Cameroon), and PSI07 (phylotype IV, Indonesia) were completely determined (Remenant et al., 2010; Xu et al., 2011; Cao et al., 2013; She et al., 2015). These genome data open up the possibilities for characterizing the global regulation mechanisms that govern the bacterial virulence, analyzing the genomic diversity within the *R. solanacearum* species complex, and may present a good opportunity to study the *R. solanacearum* evolution and the genes contributing to host-range determination. For example, recent genomic and proteomic comparisons suggested the separation of the *R. solanacearum* species complex into three species, namely the original phylotype II, phylotype IV, and the union of phylotype I and III (Prior et al., 2016). Furthermore, comparative genomic analysis have also uncovered some divergent features among closely related strains, including putative virulence effectors associated with host adaptation(Ailloud et al., 2015), and presented evidences on the horizontal gene transfer between *R. solanacearum* strains (Guidot et al., 2009).

Among the 3 strains isolated from China with complete or draft genomes available, strain FQY_4 mainly infects tobacco (Cao et al., 2013), while strains YC45 and SD54 mainly infect ginger plants (Shan et al., 2013; She et al., 2015). In May 2015, severe eggplants (*Solanum melongena* L.) wilt disease caused by *R. solanacearum* strain EP1 was occurred in Guangdong Province of China. Subsequent inoculation by drenching soil method showed that strain EP1 was also highly virulent to tomato and potato plants, and causing necrosis within 48 h and wilting about 1 week in tobacco. Here, we sequenced the complete genome of *R. solanacearum* strain EP1, and performed careful comparative analysis of EP1 and 6 other *R. solanacearum* strains with complete genome sequences available. Our analyses revealed considerable genomic divergence between these closely related strains. Particularly, we found substantial variations of key virulence genes and secretion systems. Our results suggest that the dynamic evolution of genome plays important roles during the virulence and host range change.

## Materials and methods

### Genomic DNA preparation and sequencing

*R. solanacearum* strain EP1 was grown at 28°C in casamino acid-peptone-glucose rich broth for overnight (Hendrick and Sequeira, 1984), bacterial cells were harvested by centrifugation and genomic DNA was purified using Wizard genomic DNA purification kit (Promega). The whole genome of EP1 was sequenced using a combination of PacBio with a 20 kb library (68,656 reads; >130 fold coverage) and Illumina HiSeq 2000 with a 100 bp paired end 2 k library (32,365,859 reads; >560-fold coverage). Initially, PacBio reads were assembled by SMRT Analysis 2.3.0 using the HGAP2 protocol with default parameters. Resulted contigs were validated by Illumina reads with CLC genomics workbench. Dubious regions were manually curated in CLC genomics workbench browser. Also, a separate *de novo* assembly was generated using Illumina raw reads and subsequently compared against the PacBio assembly by BLAST to identify potential plasmids of smaller sizes. The GC content calculation and gene annotation were performed using CL_GENOMICS_ (http://www.chunlab.com). The cluster of orthologous group (COG) analysis was performed to generate functional annotations for coding sequences (reference to orthologous groups, http://www.ncbi.nlm.nih.gov/COG).

### Genomic comparisons

The genome sequences of GMI1000, PO82, CMR15, FQY_4, PSI07, and YC45 were downloaded from the NCBI database. OAT (Orthologous Average Nucleotide Identity Tool) was used to measure the overall genome sequences similarity (Lee et al., 2016). Pairwise whole-genome alignments between EP1 and each of the other strains were constructed and visualized using MUMmer 3.22 (http://mummer.sourceforge.net/) with the following parameters: b, 200; c, 65; extend: 1, 20. OrthoMCL (Chen et al., 2006) cluster analyses were performed to identify the set of genes unique to strain EP1 with following parameters: *P*-value Cut-off = 1 × 10^−5^, Identity Cut-off = 90%, Percent Match Cut-off = 80%.

### Genomic islands, prophages, and CRISPRs detection

Large regions of EP1 genome have been predicted as Genomic Islands (GIs) using the interface IslandViewer 3 (Dhillon et al., 2015), executed with default parameters using GI prediction methods SIGI-HMM and IslandPath-DIMOB. Clustered regularly interspaced short palindromic repeat sequences (CRISPRs) related sequence was found in EP1 genome by using the CRISPRfinder (Grissa et al., 2007b). The presence of bacteriophage sequence was predicted using PHAST (Zhou et al., 2011). Virulence factors were predicted based on the virulence factors database (VFDB (Chen et al., 2016), http://www.mgc.ac.cn/VFs/). Type III effectors (T3es) were annotated using the IANT “Ralstonia T3E” database (Peeters et al., 2013).

## Results

### General features of *R. solanacearum* EP1 genome

**Figure 1:**
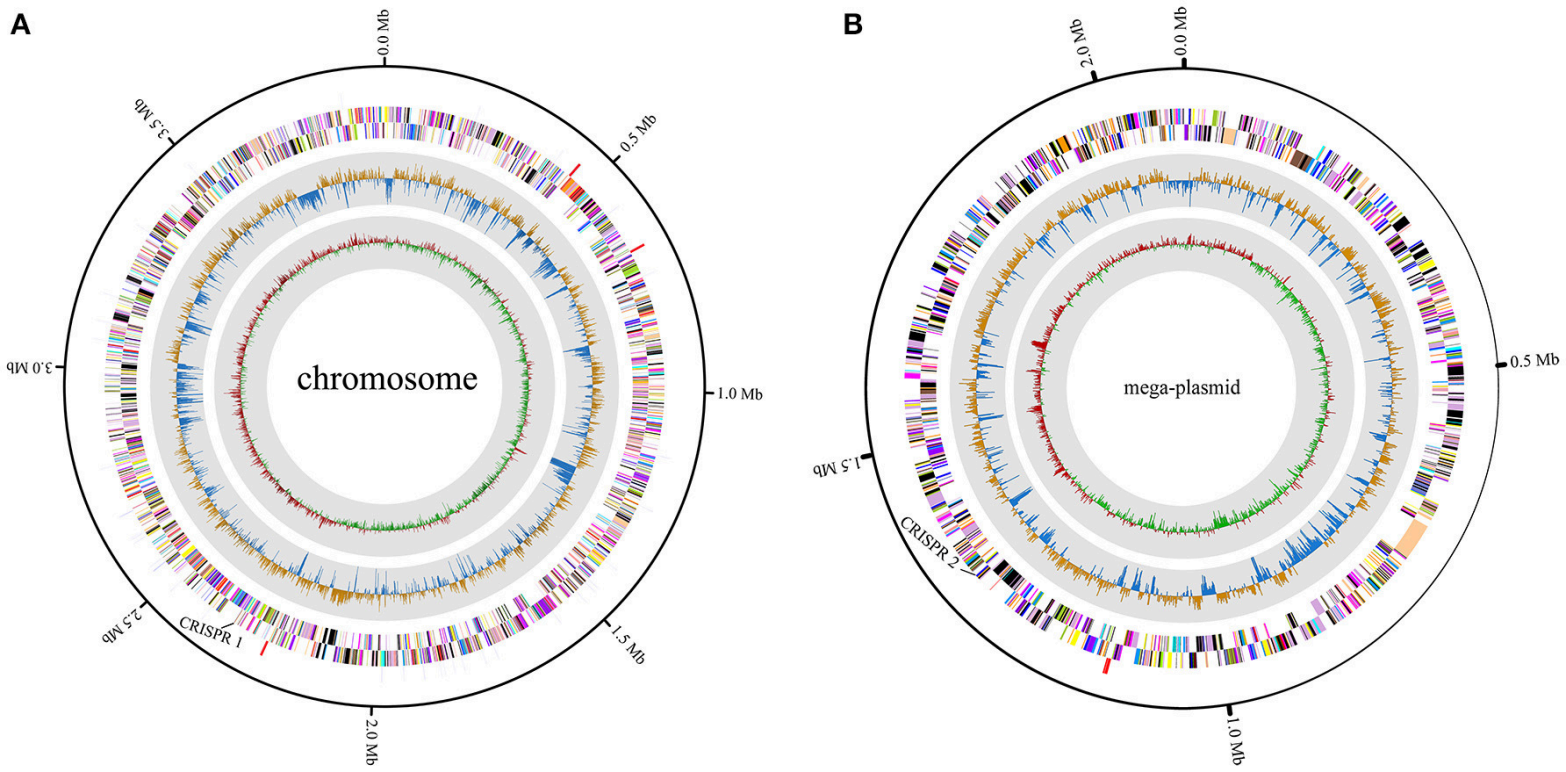
**Circular map of *R. solanacearum* strain EP1 genome. (A)** chromosome; **(B)** mega-plasmid. The distribution of the circle from outer to inner indicates rRNA and tRNA, reverse CDS, forward CDS, GC skew, and GC ratio.

**Figure 2:**
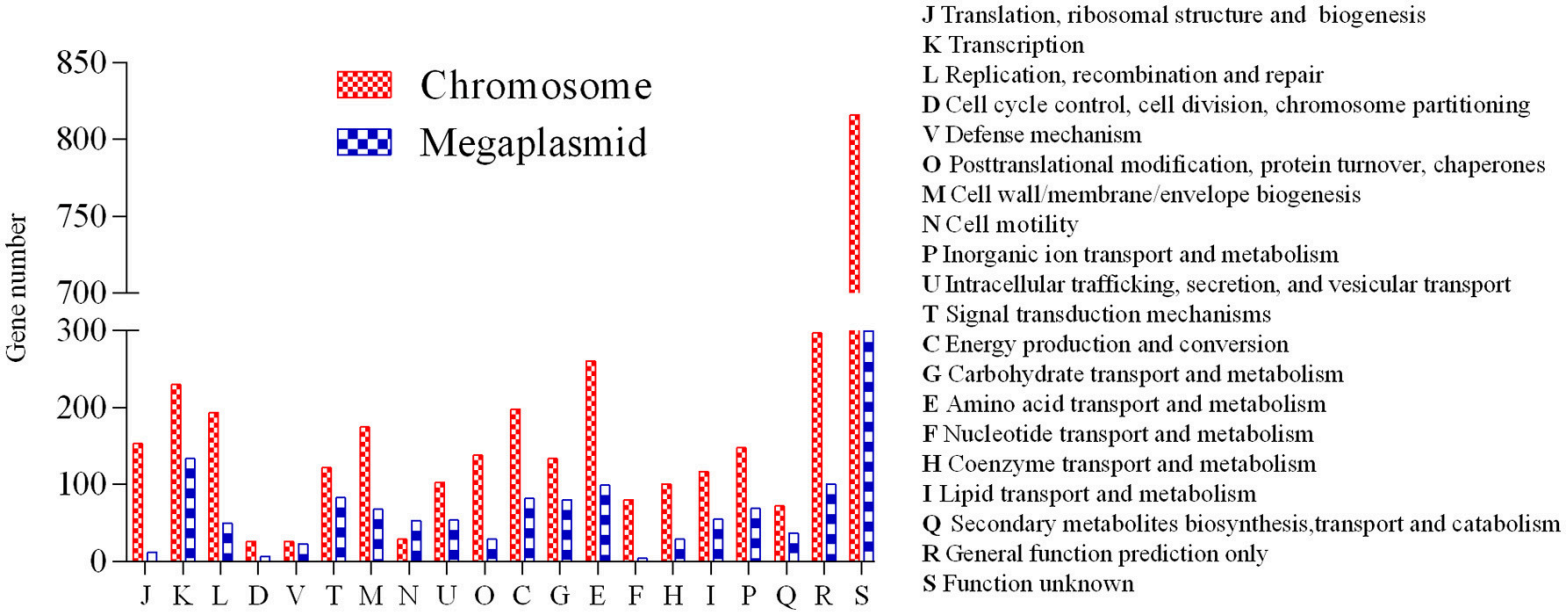
**Distribution of genes in different COG functional categories between the chromosome and the mega-plasmid in strain EP1**.

A high-quality genome assembly was generated for *R. solanacearum* strain EP1 using a combination of PacBio long read data and Illumina short read data. The assembly is 6,042,968 bp in size with a GC content of 66.72%, consisting of a circular chromosome (Figure 1A; 3,949,527 bp; GC%: 66.60%) and a mega-plasmid (Figure 1B; 2,093,441 bp; GC%: 66.94%). Neither the lysis and gel electrophoresis experiment nor the *de novo* genome assembly showed any evidence for the existence of additional plasmids. We annotated 5279 open reading frames (ORFs, hereafter referred to as genes unless otherwise specified) in the genome of strain EP1, and further assigned them to the functional categories in the COG database. In total 4869 genes were successfully classified into at least one of the 22 COG functional categories, while the remaining 410 (7.77%) genes could not be assigned with any function (Table S1). Except for the genes predicted to have general or unknown functions (1190 genes; 22.54%), the largest group of genes are involved in transcriptional roles (364 genes), followed by 360 genes responsible for amino acid transport and metabolism, 280 genes involved in energy production and conversion, 244 genes involved in replication, recombination and repair, 243 genes involved in cell wall/membrane/envelope biogenesis, 217 genes involved in inorganic ion transport and metabolism, 214 genes involved in carbohydrate transport and metabolism, and 205 genes involved in signal transduction mechanisms. Other than the protein coding genes, EP1 genome also encodes 12 rRNAs and 58 tRNAs (Table 1) which are all located on the chromosome. The chromosome and the mega-plasmid encode 3635 and 1644 genes, respectively. Comparison of the genome distribution of genes in different COG function categories showed that mega-plasmid has more genes encoding cell motility than the chromosome (Figure 2).

**Table 1:** **General genomic feature of different *Ralstonia solanacearum* strains**.

**Strain**	**Origin**	**Phylotype**	**Genome size (bp)**	**GC ratio%**	rRNA^^*^^	tRNA^*^	ORFs^*^	Putative prophage^*^	**Status**
EP1	China	I	6,042,968	66.72	12	58	5279	9	Complete
GMI1000	France	I	5,810,922	66.98	12	57	4913	7	Complete
YC45	China	I	5,732,909	67.09	6	46	4621	5	Complete
FQY_4	China	I	5,805,250	66.82	12	51	4926	7	Complete
PO82	Mexico	II	5,430,263	66.67	9	54	4577	17	Complete
CMR15	Cameroon	III	5,590,372	66.83	12	59	4748	5	Complete
PSI07	Indonesia	IV	5,605,618	66.32	9	54	4684	5	Complete

* Numbers in genome.

### Comparative genome analyses of strain EP1 to other *R. solanacearum* strains

**Figure 3:**
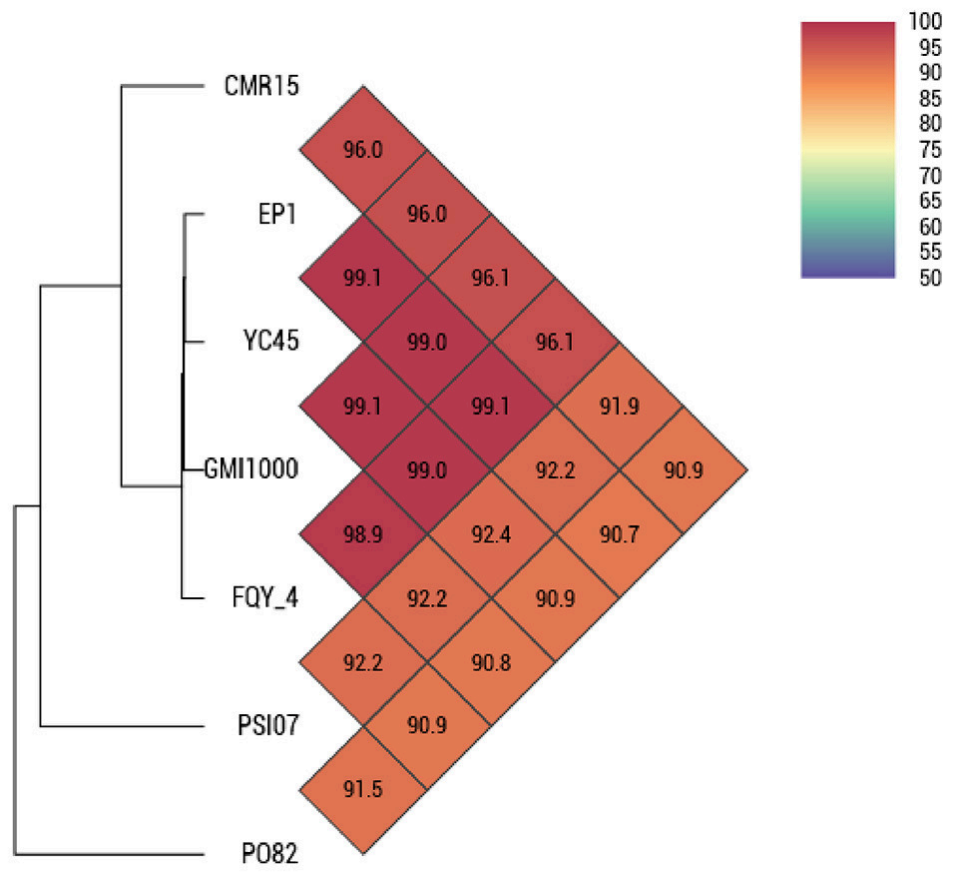
**OAT analyses on 7 complete gemomes of *R. solanacearum* strains**.

By comparing the seven complete genome sequences of *R. solanacearum* species (Table 1), we found that strain EP1 has the largest genome with 5279 ORFs, whereas strain PO82 contains the smallest genome and least number of genes (4577). The overall similarity of these genome sequences was then measured using OAT. The results showed that the four phylotype I strains are closely related, grouping together tightly in the hierarchical clustering dendrogram (Figure 3). Within the clade, strain EP1 is almost equally similar to the other 3 strains at sequence level, with the pairwise nucleotide identify (ANI) values ranging between 99.0 and 99.1%. The phylotype III strain CMR15 also shares a relatively high similarity to strain EP1 (ANI value = 96.0%) and other phylotype I strains (AVI values ≥96.0%). On the other hand, the phylotype IV strain PSI07 and phylotype II strain PO82 are more divergent from strain EP1, with ANI values of 92.2 and 90.7%, respectively (Figure 3). The GC content of these 7 genomes is similar ranging from 66.32 to 67.09% (Table 1).

**Figure 4:**
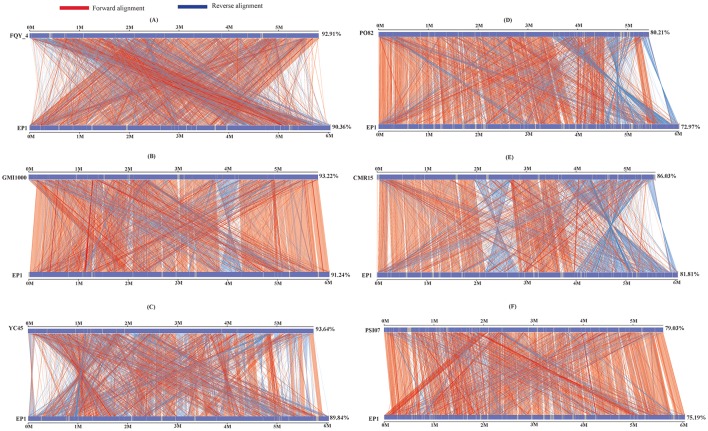
**Nucleic acid co-linearity of strain EP1 vs. strain FQY_4 (A), GMI1000 (B), YC45 (C), PO82 (D), CMR15 (E), and PSI07 (F), respectively**. The sequence of EP1 is ordered according to that of the reference bacterium based on MUMmer 3.22. The upper and following axes of co-linear graph are constructed, and pairwised nucleic acid sequence of two alignments is marked in the coordinate diagram according to its position information.

To further evaluate the genome evolution of these *R. solanacearum* strains, the genome sequence of strain EP1 was aligned with the other 6 complete genome sequences using MUMmer program. Results showed that the genome of strain EP1 is most co-linear with that of strain GMI1000 (Figure 4B), followed by strain FQY_4 with a few large inversion events (Figure 4A), and alignment with YC45 indicated a large amount of inversions distributed across the genome (Figure 4C). Among phylotype I strains, the genome sequence of strain PO82 is the least matched with strain EP1 (72.97%) with numerous gene content dissimilarities (Figure 4D). Similarly, numerous inverse fragments and dissimilarities were also found between strain EP1 and strains CMR15 and PSI07, respectively (Figures 4E,F).

**Figure 5:**
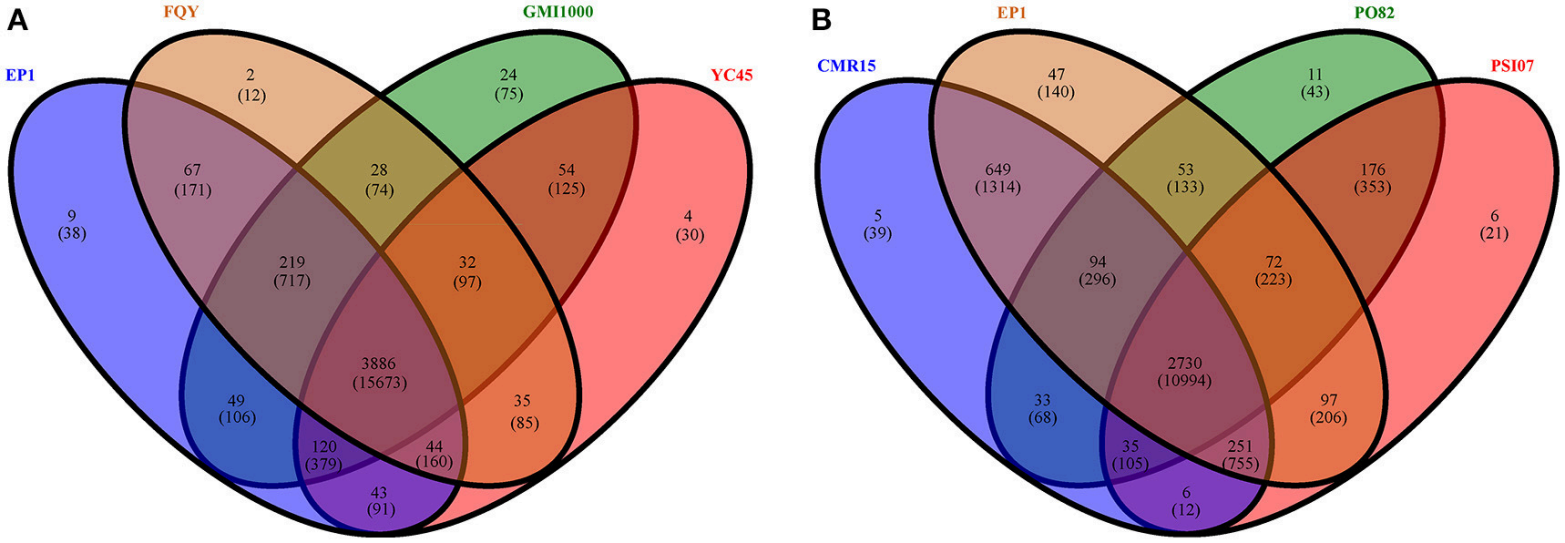
**Venn diagrams for deduced proteins of 4 phylotype I species (A), and 4 different phylotype species (B)**. The overlapping sections indicate shared numbers of deduced proteins. Values were calculated by OrthoMCL cluster analyses with the parameters: *P*-value Cut-off = 1 × 10^−5^, Identity Cut-off = 90%, Percent Match Cut-off = 80. The overlapping sections indicate shares numbers of gene families, the numbers in brackets mean the genes of the corresponding gene families.

We then further performed pan-genome analyses on the four phylotype I strains and the four phylotypes separately. In total, 4614 distinct homolog families were identified across the 4 complete genomes of phylotype I strains (EP1, FQY_4, GMI1000, and YC45). The final core genome (the gene families shared by all compared genomes) comprised 3886 gene families, accounting for 84.22% of the pan-genome (Figure 5A). Interestingly, nine gene families were found unique in strain EP1 (Figure 5A), most of which are the genes encoding hypothetical proteins, followed by those encoding insertion elements, DNA binding proteins, T3e protein AvrRpm1, putative glycine hydroxymethyltransferase, and the sel1-like repeat protein (Table S2). To investigate the pan-genome shared by different *R. solanacearum* phylotypes, we took the strain EP1 as a representative strain of phylotype I and compared with PO82 (phylotype II), CMR15 (phylotype III), and PSI07 (phylotype IV). As a result, the *R. solanacearum* pan-genome consists of 4265 gene families while the core genome is comprised of 2730 gene families, accounting for only 64.01% of the pan-genome (Figure 5B).

### Genomic islands, CRISPR and prophage sequences prediction

**Figure 6:**
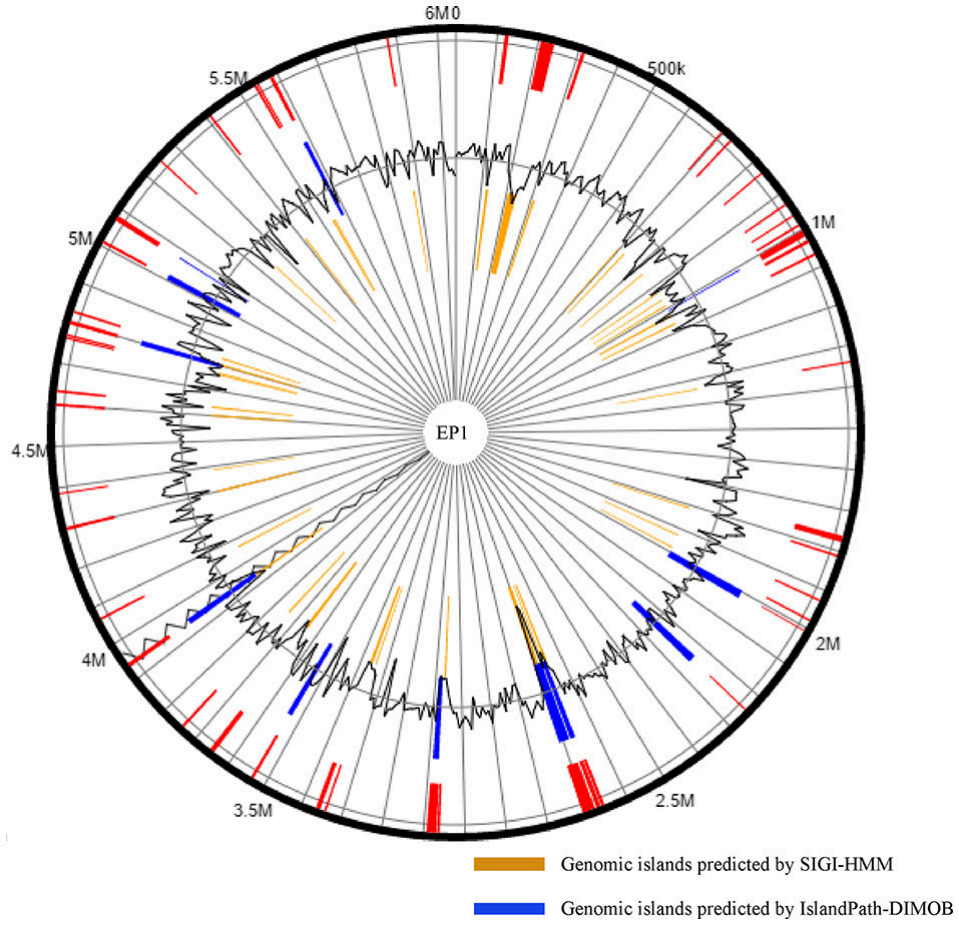
**Genomic islands distribution in strain EP1 genome**.

Existence of GI in bacteria is the evidence of horizontal origins (Langille et al., 2010). We predicted GIs in the EP1 genome using both IslandPath-DIMOB and SIGI-HMM, and methods detected in total 51 GIs. Among which 33 and 18 GIs were found on the chromosome and the mega-plasmid, respectively (Figure 6). The largest GI (31,055 bp) contains 29 genes encoding the proteins involved in replication-associated recombination protein A, putative type III restriction-modification system HindVIP enzyme subunit, modification methylase BabI, and phage proteins (Table 2, Figure 6). The other GIs predicted encode proteins related to insertion elements, transposable elements, putative prophage integrases and transposases, HTH-type transcriptional regulators, and type VI secretion system (T6SS) proteins, exoglucanase A, and oxidoreductases. Four GIs were predicted by both methods, among which the GI in between 4,796,627 and 4,803,889 was particularly variable among strains.While sequence identities of this region between EP1 and other strains were high (≥93% in all pairwise comparisons), the coverage rates were vastly different (YC45: 49%; GM1000: 100%; FQY_4: 79%; CMR15: 23%; PSI07: 44%; PO82: 23%).

**Table 2:** **Overlapping Genomic islands predicted by IslandViewer 3 methods**.

**by IslandPath-DIMOB**				**by SIGI-HMM**				**Proteins**
**GI number**	**Start**	**End**	**Size (bp)**	**GI number**	**Start**	**End**	**Size (bp)**	
5	2,666,198	2,673,859	7661	17	2,672,277	2,677,172	4895	Insertion element; Putative prophage P4 integrase; Putative transposase InsK and YkgN; HTH-type transcriptional regulator PecS
6	2,681,216	2,712,271	31,055	18	2,692,294	2,709,895	17,601	Replication-associated recombination protein A; Putative type III restriction-modification system HindVIP enzyme subunit; Modification methylase BabI; Phage proteins
9	3,936,476	3,942,888	6412	24	3,932,529	3,941,284	8755	Insertion element; Probable BsuMI modification methylase subunit YdiP; Putative type VI secretion system protein VgrGA
10	4,796,627	4,803,889	7262	32	4,799,190	4,806,223	5406	Transposable elements; Transposase; Exoglucanase A; Uncharacterized protein y4jC and oxidoreductase YbbO; HTH-type transcriptional regulator VqsM

CRISPRs can confer resistance to foreign plasmids and phages, and exist in approximately 40% of the sequenced bacterial genomes (Barrangou et al., 2007; Grissa et al., 2007a). Here, two putative CRISPR-related sequences were found in the EP1 genome using CRISPRfinder (Grissa et al., 2007b); one is located in the chromosome, and the other in the mega-plasmid (Figure 1). For comparison, the CRISPR sequences in other six completed genomes were also predicted (Table 3). The analyses unveiled two putative CRISPR sequences in the chromosome of strain GMI1000; two putative CRISPR sequence in the chromosome of strain FQY_4; one putative and one confirmed CRISPR sequences in the chromosome of strain CMR15; two confirmed CRISPR sequences in the chromosome sequence of strain PO82; two putative CRISPR sequences in the mega-plasmid of PSI07; and one putative CRISPR sequence in each of the chromosome and the mega-plasmid of strain YC45. According to the prediction results, phylotype I strains all have two CRISPRs and the sequences are completely conserved (coverage 100%, identity 100%). However, neither of them was detected in the strains PO82 and PSIO7, whereas sequence homologous to one of the CRISPR loci (1,311,176–1,311,387) in EP1 was found in the strain CMR15 chromosome. Among all strains, only YC45 shared the same distribution of the two CRISPRs as EP1 (one on each of the chromosome and mega-plasmid). Interestingly, both strains EP1 and YC45 were isolated from China, but strain EP1 belongs to phylotype I race 1 while strain YC45 belongs to race 4, and the two strains have quite different host ranges.

Phage-related sequences in bacterial genome also suggest the occurrence of horizontal gene transfer events. In strain EP1, eight bacteriophages were identified in the chromosome, of which four were intact, two were incomplete, and two were questionable (Table S4); only 1 intact bacteriophage was identified in the mega-plasmid (Table S4). One intact bacteriophage region (1,249,959–1,293,296) has a GC content of 57.40%, significantly lower than the mean GC% of the EP1 genome (66.72%), but the GC% contents of remaining eight bacteriophages are in the range of 63.56–67.36%. These bacteriophage sequences account for 4.23% of the EP1 genome. Using the same method, 17 bacteriophage genes were found in strain PO82, seven were found in both strains GMI1000 and FQY_4, and five were found in strains YC45, PSI07, and CMR15, respectively. We searched genomes of the other six strains for regions homologous to the 9 bacteriophage sequences of strain EP1; although partial matches to domain sequences of bacteriophage were found, most of their sequence coverage rates were lower than 90%.

### Genes involved in virulence

Genome annotation identified a range of well characterized *R. solanacearum* virulence genes in strain EP1, and the genes putatively involved in pathogenicity were identified (Table S5). Notably, a total of 29 bis-(3-5)-cyclic dimeric guanosine monophosphate (c-di-GMP) related genes were also identified (Table S6). Among them, 11 were located in the chromosome, including four encoding proteins with both GGDEF and EAL domains, five encoding proteins with only GGDEF domain, and only two with EAL domain; whereas 18 c-di-GMP genes were found in the mega-plasmid, including six encoding proteins with both GGDEF and EAL domains, nine with only GGDEF domain, and three with only EAL domain.

When comparing with the virulence genes of the other six *R. solanacearum* strains (Table S5), it is obvious that the gene similarities of EP1 with the other 3 phylotype I strains were higher than that with phylotype II–IV strains, though a few low-similarity genes were found among the four phylotype I strains. In general, global regulators are more conserved among these strains than other virulence genes (Table S5). There is a big group of 20 genes involved in the synthesis of hemagglutinin-related proteins. Seven of them were missing from some or even all of the other three phylotype strains (PO82, CMR15, PSI07), while the remaining 13 genes exhibited elevated levels of nucleotide sequence divergence among the seven strains. Additionally, two c-di-GMP genes (homologous to EP1 genes ORF 3789 and ORF 4062) were also not detected in strain PO82 (Table S6).

### Comparison analyses of secretion systems in *R. solanacearum* species

**Figure 7:**
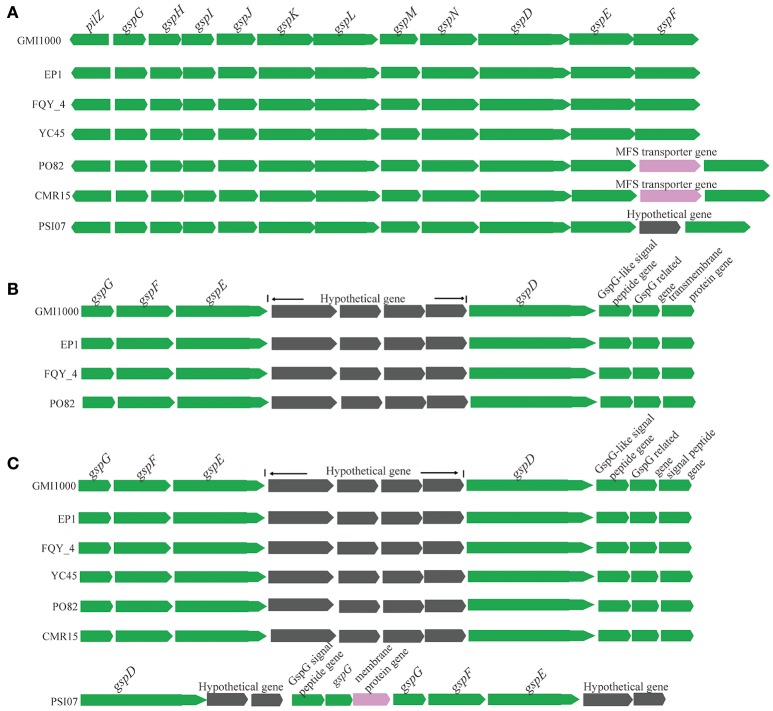
**Genetic organization of T2SS gene clusters in *R. solanacearum* species**. **(A)** The orthodox system encoded by 12 genes in the chromosome (from 375,919 to 388,223); **(B)** (from 1,297,715 to 1,307,671), and **(C)** (from 4,212,126 to 4,221,456) are two unorthodox T2SS.

The genes encoding four important secretion systems (type II, III, IV, and VI systems) in the seven completely sequenced *R. solanacearum* species were compared in this study. Similar with strain GMI1000 (Genin and Boucher, 2004), EP1 possesses three type II secretion systems (T2SS) (Figure 7). The first one is the orthodox system encoded by 12 genes in the chromosome (from 375,919 to 388,223). This gene cluster is highly conserved and shares a high similarity with strains GMI1000, YC45 and FQY_4 (coverage 100%, identity 99%), CMR15 (coverage 100%, identity 95%), PSI07 (coverage 99%, identity 95%), and PO82 (coverage 99%, identity 94%). In strains CMR15 and PO82, one MFS transporter gene and one hypothetical protein gene was found inserted between *gspE* and *gspF*, respectively (Figure 7A). The other two T2SS are unorthodox systems, one possessing seven core genes (ranged from 1,297,715 to 1,307,671) in EP1, which was only found in strains GMI1000 (coverage 100%, identity 99%), FQY_4 (coverage 100%, identity 99%), and PO82 (coverage 99%, identity 94%) with four hypothetical genes inserted between *gspE* and *gspD* (Figure 7B). The other unorthodox T2SS contains seven core genes (from 4,212,126 to 4,221,456) located in the mega-plasmid, sharing a high similarity with strains GMI1000, YC45, and FQY_4 (coverage 100%, identity 99%), CMR15 (coverage 99%, identity 97%), and PO82 (coverage 99%, identity 94%), while distinct gene rearrangements were found in the counterpart region of strain PSI07 (Figure 7C).

**Figure 8:**
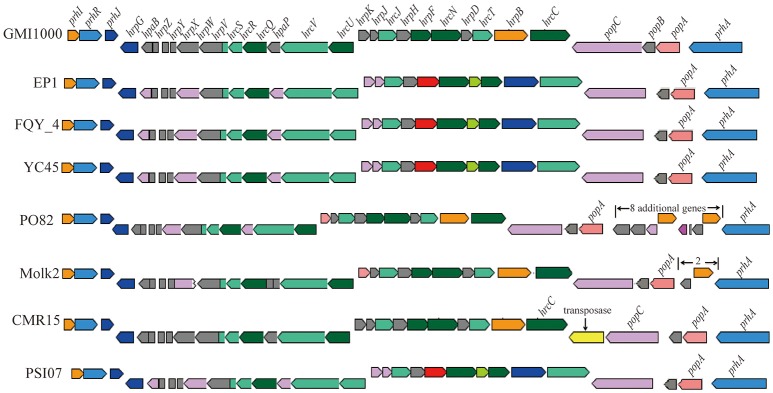
**Genetic organization of *hrp* clusters in *R. solanacearum* species**.

We further analyzed the *hrp* gene cluster of type III secretion system (T3SS) in these *R. solanacearum* strains (Figure 8), which is the key virulence determinant conserved in many different bacterial species (He et al., 2004). In strain EP1, the *hrp* gene cluster is located in the mega-plasmid and spans 29.681 kb (from 5,205,009 to 5,234,690), composed of 30 genes. Comparison of the *hrp* clusters of strain EP1 and other *R. solanacearum* strains showed that the *hrp* cluster is conserved among the phylotype I strains, sharing high similarity with strains GMI1000 and FQY_4 (coverage 100%, identity 99%), as well as YC45 (coverage 89%, identity 99%); the similarity with PSI07 (coverage 99%, identity 91%), CMR15 (coverage 89%, identity 96%), and PO82 (coverage 93%, identity 91%). By aligning the sequences of the *hrp* cluster (Figure 8), one putative transposase was found between *hrcC* and *popC* in strain CMR15, and eight additional genes were inserted between *prhA* and *popA* in strain PO82. Similar events were also detected in the other strains with draft genome assemblies; for example, two genes with unknown function were inserted between *prhA* and *popA* in strain Molk2.

A total of 71 T3es were found in EP1 genome (Table S7), among of which 65 T3es were also present in the other three phylotype I strains, accounting for 69.15% (65/94) of their pan-effectorome. A total of 110 T3es were found among strains of the four different phylotype (EP1, CMR15, PSI07, and PO82), only 48 (43.64%) of which belongs to the core-genome. Moreover, considering that the host ranges of the strains are substantially different, for the strain EP1 mainly infect on the solanaceae crops, while the strain YC45 only infect the ginger (She et al., 2015), we performed further comparison between them using the T3e online database. The results showed that the three T3es (RipC2, RipT, RipAL) were present in strain EP1 but were absent in strain YC45, while the two T3es (RipE2, RipF2) were absent in strain EP1 but were present in strain YC45. Taking the 23 T3es with defined roles in strain GMI1000 as references (Coll and Valls, 2013), the corresponding homolog in other six completed *R. solanacearum* genomes were compared and analyzed (Table 3). Strain EP1 also contains 23 effector genes which are all highly similar to the counterparts of strain GMI1000 with similarity higher than 94%. However, in the other two phylotype I strains FQY_4 and YC45, the homolog of effector *RSc0826* was absent. Substantially higher levels of divergence were found in strains PO82 (phylotype II), CMR15 (phylotype III), and PSI07 (phylotype IV). Except for a few highly conserved effectors, the identities of most effectors were lower than 90% compared with their counterparts in GMI1000, and up to seven effector genes were absent in some or all of the three strains.

**Table 3:** **Coverage (%)/Identity (%) comparison with defined role T3es genes of *R. solanacearum* strain GMI1000**.

**GMI1000**		**Phylotype I**			**Phylotype II**	**Phylotype III**	**Phylotype IV**
**Gene**	**Protein**	**EP1**	**YC45**	**FQY_4**	**PO82**	**CMR15**	**PSI07**
*RSc0608*	AvrA	100/99	100/100	100/97	75/76	100/98	80/77
*RSp0822*	AvrPphF	100/99	100/99	100/97	99/70	96/93	Absent
*RSc2139*	Awr1	100/99	100/99	100/99	Absent	Absent	Absent
*RSp0099*	Awr2	100/99	100/99	100/99	100/83	100/95	100/85
*RSp0847*	Awr4	100/99	100/99	100/99	100/83	100/94	100/86
*RSp1024*	Awr5	100/99	100/99	100/99	97/86	100/96	98/88
*RSp0914*	Gala1	100/99	100/99	100/99	47/67	71/67	100/93
*RSp0028*	Gala3	100/98	100/98	100/98	95/74	100/91	99/79
*RSc1801*	Gala5	100/99	100/99	100/99	99/77	100/93	99/82
*RSc1356*	Gala6	100/98	100/87	100/87	92/81	100/85	98/83
*RSc1357*	Gala7	99/99	100/99	100/98	96/76	99/77	93/85
*RSp0877*	PopA	100/98	100/96	100/95	100/79	99/88	93/84
*RSp0842*	–	100/99	100/99	100/99	100/90	100/97	100/93
*RSc0826*	PopP1	100/94	Absent	Absent	100/94	Absent	Absent
*RSc0868*	PopP2	100/99	100/99	100/99	100/81	100/79	Absent
*RSc1839*	Skwp4	100/99	100/99	100/99	97/82	100/89	93/74
*RSc1815*	Rip19	100/99	100/99	100/98	Absent	Absent	84/77
*RSp0304*	Rip34	100/99	100/94	100/98	100/76	100/77	100/90
*RSp0732*	Rip39	100/98	100/99	100/98	100/91	100/92	Absent
*RSp1281*	Rip64	100/99	100/99	100/99	95/87	99/94	95/88
*RSc0257*	Rip3	100/99	100/99	98/75	99/85	99/93	99/80
*RSp1022*	Rip55	100/98	100/97	100/98	96/72	100/88	95/74
*RSc2359*	Rip23	100/99	100/99	100/99	Absent	Absent	Absent
Identity (Id)		Id ≥ 90%	80% ≤ Id < 90	70% ≤ Id < 80	60% ≤ Id < 70	Absent	

Similarly, we took the genome of strain GMI1000 as reference to search for the counterparts of the type IV secretion system (T4SS) gene cluster (*RSc2574–RSc2588, RSp0179,* and *RSp1521*; Salanoubat et al., 2002; Genin and Boucher, 2004). Except for strains FQY_4 and Molk2 which have all the 17 homologous T4SS genes, strain CMR15 only has five T4SS genes (homologous to *RSc2575, RSc2576, RSc2586, RSp0179*, and *RSp1521*), and strains EP1, PSI07, PO82, and YC45 contain only 3 T4SS genes (homologs to *RSc2575, RSp0179*, and *RSp1521*).

**Figure 9:**
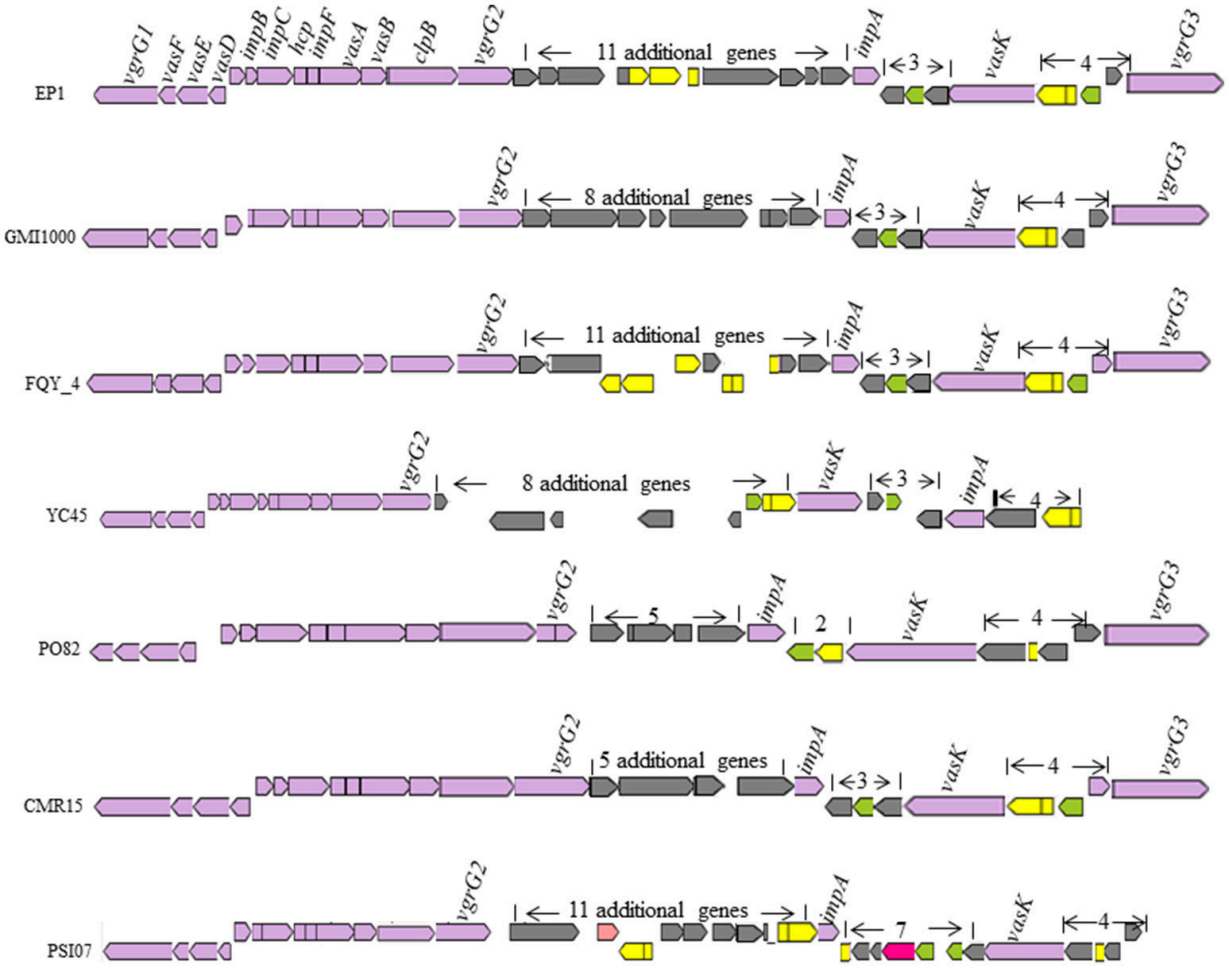
**Genetic organization of T6SS clusters in *R. solanacearum* species**.

T6SS is widely spread among gram negative bacteria (Records, 2011). In strain GMI1000, this secretion system apparatus contains an approximate 42-kb region in the mega-plasmid (Leiman et al., 2009). In strain EP1, the T6SS locus spans 47.927 kb with 16 core genes required for synthesis of this system, 11 additional genes were found inserted between *vgrGA2* and *impA*, three additional genes inserted between *impA* and *vasK*, and four additional ORFs inserted between *vasK* and the last gene *varGA3* (Figure 9). The core T6SS genes in these chosen strains were conserved, while the inserted sequences (5–11 additional genes) between *varGA2* and *impA* varied significantly. The whole T6SS gene cluster of strain EP1 shares high similarity with strains GMI1000 (coverage 93%, identity 99%), FQY_4 (coverage 83%, identity 99%), YC45 (coverage 90%, identity 99%), PO82 (coverage 90%, identity 93%), CMR15 (coverage 81%, identity 95%), and PSI07 (coverage 79%, identity 94%). In strain YC45, gene rearrangement was found where two genes between *vasK* and *impA* were inversed relative to other strains.

## Discussion

*R. solanacearum* species complex is considered one of the best models to understand the micro- and macro-evolution patterns leading to the formation of emerging ecotypes adapting to local environmental conditions (Genin and Denny, 2012). In this study, we generated the complete genome sequence of strain EP1 using a combination of PacBio and Illumina HiSeq 2000 sequencing techonologies, and performed genome-wide comparisons between EP1 and six other *R. solanacearum* strains representing four different phylotypes. Our results provide further evidences that the genome rearrangement, gene deletion and insertion, and other genomic variations are frequent during the evolution course of *R. solanacearum* species. Complete sequencing of the strain EP1 genome paves the way for further identification and characterization of the genetic elements and mechanisms that contribute to bacterial virulence and host specificity, and provides insight into virulence factors variance and genome diversities.

Results of OAT and MUMmer analyses showed that the genome of strain EP1 is more similar with that of the three phylotype I strains (FQY_4, GMI1000, YC45) than the three strains from other phylotypes (PO82, CMR15, and PSI07). Notably, the ANI value between strain CMR15 (phylotype III) and the three phylotype I strains were higher than 96%. According to the taxonomic standard that the strains with ANI >95% are considered as belonging to the same species (Goris et al., 2007; Kim et al., 2014), the strain CMR15 shall belong to the same group with the four phylotype I strains, consistent with a recent literature (Prior et al., 2016). In addition, though the highest genome similarity exists between strains EP1 and YC45 (99.1%, Figure 3), large amount of gene rearrangement events were unveiled between these two strains by the MUMmer analysis (Figures 4A–C). The notion is further strengthened by the fact that numerous inversions and gene deletion/insertion events were also found when comparing strain EP1 with other three different phylotype strains (PO82, CMR15, and PSI07; Figures 4D–F). Ancestral state reconstruction analyses have suggested that *R. solanacearum* was originated from Oceania/Indonesia region (phylotype IV strains, such as strain PSI07; Wicker et al., 2012). According to this hypothesis, the phylotype I strains, which have genomes larger than PSI07, might have evolved from phylotype IV strains through mechanisms leading to increased genome size. On the other hand, phylotype II strain PO82 and phylotype III strain CMR15 were derived through mechanisms resulting in decreased genome size as their genome sizes are smaller than those of other phylotype strains (Table 1). Whether this is the general trend of phylotype evolution or isolated cases awaits further verification with further large scale genome sequencing analysis. Nevertheless, the findings from this study showed that *R. solanacearum* strains are experiencing highly dynamic genome evolution, which likely has great importance in the adaptation of *R. solanacearum* species to new host plants and different environmental conditions.

Core- and pan-genome analyses of *R. solanacearum* species showed that the percentage of gene families belonging to the core-genome is higher within phylotype I (84.22%) compared to between phylotypes (64.01%), indicating a higher level of genetic conservation among strains of the same phylotype (Figure 5). Among the four phylotypes, the core genome represents about 2730 gene families, which is a smaller percentage of the total than that of *Pseudomonas syringae* species (~3400 core genes), although their genomes are almost equivalent in size (Baltrus et al., 2011). It has been reported that the genome of *R. solanacearum* has a mosaic structure (Salanoubat et al., 2002), which makes it easy for *R. solanacearum* species to acquire exogenous DNA (Bertolla et al., 1997; Nakamura et al., 2004; Coupat-Goutaland et al., 2011). As the same time, the pre-existing genes could be lost by mutation (Ochman and Moran, 2001). Consistent with its largest genome size among the strains included in this study, strain EP1 contains ten unique insertion element genes, further supporting that the insertion elements play an important role in the evolution of bacterial genome, and may have contributed to the apparent diversity among *R. solanacearum* species. Previous results have demonstrated that bacteriophages are double-edged swords, they could either enhance or repress the virulence of *R. solanacearum* species and thus affect the outcome of pathogen-host interactions (Addy et al., 2012a,b). Similarly, the number and distribution of CRISPRs and bacteriophage sequences also differ in the strains of different phylotypes (Table S3, Table S4). Therefore, the variable capacity of different strains in conferring resistance to foreign genetic elements and the insertion of exogenous genes may also contribute to genome variations of these strains.

The bipartite genome made *R. solanacearum* species exercise in a very ingenious regulation way to adapt to various host plants and different life styles. Evidence indicates that the mega-plasmid was originated from a dispensable plasmid (Salanoubat et al., 2002), and gradually evolved to an indispensable component of the genome during long-term evolution (Genin and Boucher, 2004). Comparison of function categories between chromosome and mega-plasmid in EP1 showed patterns consistent with the previous report that both replicons contain growth essential genes (Salanoubat et al., 2002), and many important virulence genes are located in the mega-plasmid, such as the *hrp* clusters, 18 c-di-GMP genes, numerous flagella genes, and T6SS genes. In addition, the OAT analysis on the chromosomes and the mega-plasmids showed that the sequence similarities between the mega-plasmids are generally lower than that between the chromosomes in the chosen strains (Figure S1), suggesting that the mega-plasmid evolves more rapidly among the *R. solanacearum* strains. Apparently, existence of mega-plasmid increases the complexity and spectrum of *R. solanacearum* genome evolution.

In addition to the aforementioned genome-wide comparisons, we also conducted thorough investigations on the variation of the genes encoding key virulence factors which play significant roles in pathogenesis and bacterial proliferation in host plants, such as global regulators, exopolysaccharide biosynthesis, hydrolytic enzymes, adhesion proteins, pilus, and fimbrial biogenesis proteins, transmembrane proteins, toxins, resistance proteins to oxidative stress, plant hormones, signaling molecules, and secretion systems (Buell et al., 2003). Based on our results, most of these pathogenicity-related genes are conserved among the phylotype I strains, while significant gene variations and even gene losses were found among different phylotype strains. For instance, the genes encoding hemagglutinin-related proteins were found substantially variable among the *R. solanacearum* strains included in this study (Table S5). The hemagglutinin related proteins are commonly found in bacterial organisms, which contribute to attachment and aggregation of bacterial cells on host surface or tissues (Jacob-Dubuisson et al., 2001; Van Sluys et al., 2002). Whether these variations could affect the ability of bacterial attachment and aggregation, or even be involved in pathogen-host interaction is worthy of further investigations.

Although, the GMI1000 genome contains genetic information for the expression of all six major secretion systems (Salanoubat et al., 2002), only a limited number of them have been characterized functionally. In this study, four well known secretion systems were compared among the *R. solanacearum* strains with complete genome sequences. Based on the alignment analysis of the gene clusters, T2SS cluster sequences of all these *R. solanacearum* strains have a high coverage and identity, except for the gene insertions and gene rearrangement occurred in some strains (PO82, CMR15, and PSI07; Figure 7). Similarly, we have analyzed the T4SS which is known for translocation of genetic materials and effector proteins into host cytosol or other bacterial cells (Burns, 2003; Angot et al., 2007; Guidot et al., 2009), and results showed that except that strain GMI1000 and FQY_4 contains a set of 17 T4SS genes, the remaining five strains keep only 3–5 genes, suggesting the T4SS may be largely degenerated during the course of bacterial evolution. As T4SS degeneration occurred in all the four phylotype strains, we reason that T4SS may not play a vital role in the virulence and host range specificity of the *R. solanacearum* species.

T3SS is one of the widely conserved key virulence determinants that could inject a number of effector proteins into host cells to influence host physiological status and signaling mechanisms (Bauer et al., 1994; Lindgren, 1997; Coll and Valls, 2013). Interesting variations were found in the T3SS of *R. solanacearum* strains. Previous studies showed that the *hrp* gene cluster of *R. solanacearum* which encodes the T3SS related proteins, plays a critical role in bacterial virulence and determination of host range specificity (Poueymiro et al., 2009; Lohou et al., 2014). Sequence alignment showed that the *hrp* cluster was highly conserved in EP1, GMI1000, YC45, PSI07, and FQY_4, while some variations were found in other strains, such as the transposase gene insertion in strain CMR15 and other genes insertion in strains PO82 and Molk2 (Figure 8). In contrast, substantial variations in T3es genes were found among these strains. The comparison of the 23 T3es with defined roles showed that the T3es genes from phylotypes II, III, and IV strains were quite different from phylotype I strains at levels of both gene copy number and sequence conservation (Table 3). For instance, strain EP1 has three copies of the unique effector gene (Table S2), which encodes a homolog of the well characterized effector protein AvrRpm1. In *P. syringae*, AvrRPm1 is known for its activity in suppression of host basal defenses induced by microbe-associated molecular patterns when not recognized by an cognate R-protein (Kim et al., 2009). Therefore, these extra copies of the unique effector gene in strain EP1 might hold the key to decipher the mechanisms underpinning the observed strong virulence phenotype and broad host range specificity of this pathogen. Extensive studies have showed that T6SS could contribute to pathogenicity, host colonization, and mediate biofilm formation (Mougous et al., 2006; Hood et al., 2010; Zhang et al., 2014). Two to eleven additional genes were found inserted in the T6SS gene clusters of some strains, including the genes encoding membrane proteins, transposases and quite a few hypothetical proteins. Additionally, gene inversion was also detected in the T6SS cluster. Given the general important roles of T6SS in pathogens, these variations in the T6SS gene cluster may likely cause changes in bacterial pathogenicity and the capability of host colonization.

Taken together, the complete genome sequence of strain EP1 represents an essential resource and platform for subsequent analysis of the pathogenic mechanisms of this highly virulent pathogen that causes significant damages on plantation of eggplant and other solanaceous crops in China. In addition, comparative genomic analysis of seven complete genome sequences of *R. solanacearum* strains provides novel insights into the diversity and evolution of *R. solanacearum* genome, as well as useful clues on potential genetic mechanisms which may cause variations in host range specificity and virulence of different *R. solanacearum* strains. Furthermore, our results also suggest that some unique genes detected in strain EP1 may likely play roles in pathogen-host interaction. These findings would certainly facilitate further studies on this important pathogen and for developing new strategies on disease control and prevention.

### Ethics statement

All the experiments were performed according to the experiment security regulations of South China Agricultural University (SCAU), and approved by the biosafety committee in SCAU.

### Data accessibility

The whole genome sequence of *R. solanacearum* EP1 has been deposited at GenBank under the accession number CP015115 (chromosome) and CP015116 (mega-plasmid). The strain EP1 is accessible in Guangdong Province Key Laboratory of Microbial Signals and Disease Control, South China Agricultural University, People's Republic of China and in Collection Française des Bactéries Phytopathogènes, France, accession no. CFBP8480.

## Author contributions

PL contributed to annotation of the genome, PL, LZ designed the experiments and wrote the paper, DW, JY helped to extract the DNA, YD, ZJ, BC, and ZH helped to isolate the *R. solanacearum* strain, JZ, LZ revised the manuscript.

## Funding

This work was supported by the National Basic Research and Development Program (973 Program, grant number 2015CB150600), China Postdoctoral Science Foundation (2015M572329), Pearl River Nova Program of Guangzhou (No. 201506010067).

### Conflict of interest statement

The authors declare that the research was conducted in the absence of any commercial or financial relationships that could be construed as a potential conflict of interest.

## Supplementary material

Table S1 **Functional categories based on COG in EP1**.

Table S2 **Nine unique gene families in strain EP1**.

Table S3 **CRISPRs sequences loci in the genome of *R. solanacearum* strains**.

Table S4 **Bacteriophage sequences information in *R. solanacearum* strain EP1**.

Table S5 **Comparison analysis of known and candidate virulence factors in the 7 completely sequenced *R. solanacearum* strains**.

Table S6 **c-di-GMP genes in *R. solanacearum* strain EP1**.

Table S7 **The annotated T3es using the IANT “Ralstonia T3E” database**.

Figure S1 **OAT analysis of the chromosome and the mega-plasmid sequences among the 7 completely sequenced *R. solanacearum* species**.
