# Supplementary material for: Genomic Analysis of Phylotype I Strain EP1 Reveals Substantial Divergence from Other Strains in the Ralstonia solanacearum Species Complex
Source: Front Microbiol. 2016 Oct 26;7:1719. doi: 10.3389/fmicb.2016.01719 (PMC5080846; doi:10.3389/fmicb.2016.01719)
Supplement: Table S1 — Functional categories based on COG in EP1. [file Table1.PDF]

SI1 Functional categories based on COG in EP1

Description	Number of Genes	(%)
General function prediction only	398	7.54
Transcription	364	6.89
Amino acid transport and metabolism	360	6.82
Energy production and conversion	280	5.30
Replication, recombination and repair	244	4.62
Cell wall/membrane/envelope biogenesis	243	4.60
Inorganic ion transport and metabolism	217	4.11
Carbohydrate transport and metabolism	214	4.05
Signal transduction mechanisms	205	3.88
Lipid transport and metabolism	172	3.26
Posttranslational modification, protein turnover, chaperones	167	3.16
Translation, ribosomal structure and biogenesis	165	3.13
Intracellular trafficking, secretion, and vesicular transport	157	2.97
Coenzyme transport and metabolism	130	2.46
Secondary metabolites biosynthesis, transport and catabolism	109	2.06
Nucleotide transport and metabolism	84	1.59
Cell motility	82	1.55
Defense mechanisms	49	0.93
Cell cycle control, cell division, chromosome partitioning	33	0.63
RNA processing and modification	3	0.057
Chromatin structure and dynamics	2	0.038
Extracellular structures	1	0.019
Function unknown	1190	22.54
Not in COG	410	7.77
	5279	100
