# Supplementary material for: Genomic Analysis of Phylotype I Strain EP1 Reveals Substantial Divergence from Other Strains in the Ralstonia solanacearum Species Complex
Source: Front Microbiol. 2016 Oct 26;7:1719. doi: 10.3389/fmicb.2016.01719 (PMC5080846; doi:10.3389/fmicb.2016.01719)
Supplement: Table S2 — Nine unique gene families in strain EP1. [file Table2.PDF]

SI2 Nine unique gene families in strain EP1

Unique gene family	Gene ID in EP1	Function annotation
1	2666, 4321, 4364	DNA binding protein
2	156, 351, 1258, 2311,2741, 2814, 3056, 3453, 4441, 4486	Insertion element IS402 uncharacterized 162 kDa protein
3	308, 2687	hypothetical protein
4	1086, 1675	hypothetical protein
5	2693, 2824	hypothetical protein
6	859, 865, 4386	type III effector protein AvrRpm1
7	155, 352, 1259, 2312, 2740,2815, 3057, 3454, 4440, 4485	hypothetical protein
8	2733, 2802, 2805, 4307	putative Glycine hydroxymethyltransferase
9	4433, 4435	Sel1-like repeat protein
