# Supplementary material for: Genomic Analysis of Phylotype I Strain EP1 Reveals Substantial Divergence from Other Strains in the Ralstonia solanacearum Species Complex
Source: Front Microbiol. 2016 Oct 26;7:1719. doi: 10.3389/fmicb.2016.01719 (PMC5080846; doi:10.3389/fmicb.2016.01719)
Supplement: Table S3 — CRISPRs sequences loci in the genome of R. solanacearum strains. [file Table3.DOCX]

SI3 CRISPRs sequences locus in the genome of *R. solanacearum* strains

| Strain | Chromosome | | Mega-plasmid | |
| --- | --- | --- | --- | --- |
|  | Confirmed | Putative | Confirmed | Putative |
| CMR15 | 663,117-663,309 | 2,521,192-2,521,299 | ND |  |
| EP1 | ND | 2,335,414-2,335,524 | ND | 1,311,176-1,311,387 |
| GMI1000 |  | 1,445,581-1,445,691 | ND |  |
|  |  | 2,489,798-2,498,026 |  |  |
| FQY_4 |  | 1,459,682-1,459,792 | ND |  |
|  |  | 2,437,870-2,438,081 |  |  |
| PO82 | 1,127,636-1,128,517 | ND | ND |  |
|  | 1,137,444-1,138,449 | ND | ND |  |
| PSI07 | ND | ND | ND | 425,757-425,917 |
|  | ND | ND | ND | 684,623-684,702 |
| YC45 | ND | 2,280,319-2,280,429 | ND | 797,467-797,577 |

ND, no detected.
