# Supplementary material for: Genomic Analysis of Phylotype I Strain EP1 Reveals Substantial Divergence from Other Strains in the Ralstonia solanacearum Species Complex
Source: Front Microbiol. 2016 Oct 26;7:1719. doi: 10.3389/fmicb.2016.01719 (PMC5080846; doi:10.3389/fmicb.2016.01719)
Supplement: Table S4 — Bacteriophage sequences information in R. solanacearum strain EP1. [file Table4.PDF]

SI4 Bacteriophage sequences information in EP1 genome

No.	Length (Kb)	Completeness	CDS	Position	Possible phage	GC ratio%
1	12.7	questionable	17	227643-240342	PHAGE_Synech_S_CBS3_NC_015465	67.36
2	28.9	questionable	20	245371-274316	PHAGE_Stenot_S1_NC_011589	66.37
3	43.5	intact	43	1099477-1143047	PHAGE_Ralsto_phiRSA1_NC_009382	63.86
4	43.3	intact	29	1249959-1293296	PHAGE_Ralsto_RS603_NC_025454	57.40
5	45.8	intact	55	1682638-1728528	PHAGE_Ralsto_RSY1_NC_025115	64.58
6	21.8	intact	29	1947171-1968994	PHAGE_Burkho_KS9_NC_013055	66.38
7	19.2	incomplete	18	2859877-2879153	PHAGE_Stenot_S1_NC_011589	66.26
8	15.9	incomplete	11	2936721-2952626	PHAGE_Shewan_1/44_NC_025463	64.25
9*	24.5	intact	37	2068261-2092808	PHAGE_Burkho_KS10_NC_011216	63.56

* means the bacteriophage sequence is located in the mega-plasmid.
