# Supplementary figures and images for: Genomic Analysis of Phylotype I Strain EP1 Reveals Substantial Divergence from Other Strains in the Ralstonia solanacearum Species Complex
Source: Front Microbiol. 2016 Oct 26;7:1719. doi: 10.3389/fmicb.2016.01719 (PMC5080846; doi:10.3389/fmicb.2016.01719)

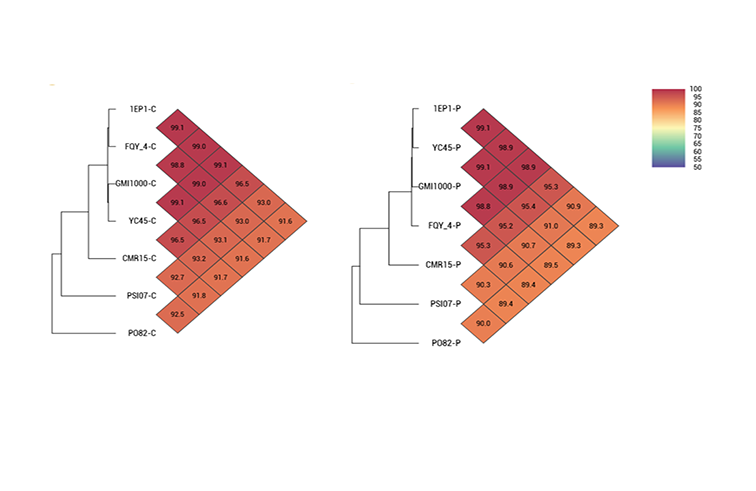

Supplement: Figure S1 — OAT analysis of the chromosome and the mega-plasmid sequences among the 7 completely sequenced R. solanacearum species. [file Image1.TIF]
